# Supplementary material for: Hot spot prediction in protein-protein interactions by an ensemble system
Source: BMC Syst Biol. 2018 Dec 31;12(Suppl 9):132. doi: 10.1186/s12918-018-0665-8 (PMC6311905; doi:10.1186/s12918-018-0665-8)
Supplement: Supplementary file 2 — All relevant MATLAB code. A simple Matlab implement of our predictor. (ZIP 19,742 kb) [file 12918_2018_665_MOESM2_ESM.zip › Additional file 2/Read ASA/ASA website/dbMPIKT/4GU0E.html]

 NetSurfP - Protein Surface Accessibility and Secondary Structure Predictions

  

|  |  |  |
| --- | --- | --- |
|  |  | NetSurfP - Protein Surface Accessibility and Secondary Structure Predictions  Technical University of Denmark |

  


---

```
# For publication of results, please cite:
# A generic method for assignment of reliability scores applied to solvent accessibility predictions.
# Bent Petersen, Thomas Nordahl Petersen, Pernille Andersen, Morten Nielsen and Claus Lundegaard
# BMC Structural Biology 2009, 9:51 doi:10.1186/1472-6807-9-51
#
# Column 1: Class assignment - B for buried or E for Exposed - Threshold: 25% exposure, but not based on RSA
# Column 2: Amino acid
# Column 3: Sequence name
# Column 4: Amino acid number
# Column 5: Relative Surface Accessibility - RSA
# Column 6: Absolute Surface Accessibility
# Column 7: Z-fit score for RSA prediction
# Column 8: Probability for Alpha-Helix
# Column 9: Probability for Beta-strand
# Column 10: Probability for Coil
E A  4GU0E                  1    0.882  97.185  -0.326   0.003   0.003   0.994
E R  4GU0E                  2    0.564 129.064  -0.111   0.268   0.043   0.689
E T  4GU0E                  3    0.523  72.540  -0.261   0.455   0.046   0.498
E M  4GU0E                  4    0.347  69.395  -0.299   0.622   0.015   0.363
E Q  4GU0E                  5    0.512  91.407   0.112   0.717   0.014   0.269
E T  4GU0E                  6    0.380  52.734  -0.067   0.717   0.014   0.269
B A  4GU0E                  7    0.240  26.415  -1.339   0.751   0.050   0.199
E R  4GU0E                  8    0.384  87.959  -0.414   0.717   0.014   0.269
E K  4GU0E                  9    0.572 117.660  -0.378   0.717   0.014   0.269
E S  4GU0E                 10    0.521  61.073  -1.378   0.522   0.016   0.462
E T  4GU0E                 11    0.430  59.710  -1.375   0.257   0.016   0.727
E G  4GU0E                 12    0.578  45.481  -2.521   0.058   0.017   0.925
E G  4GU0E                 13    0.446  35.069  -1.690   0.058   0.017   0.925
E K  4GU0E                 14    0.544 111.839  -0.385   0.268   0.043   0.689
B A  4GU0E                 15    0.277  30.536  -0.765   0.257   0.016   0.727
B P  4GU0E                 16    0.279  39.661  -0.706   0.430   0.016   0.555
E R  4GU0E                 17    0.322  73.646  -0.430   0.694   0.003   0.303
E K  4GU0E                 18    0.498 102.500  -0.127   0.802   0.014   0.185
E Q  4GU0E                 19    0.460  82.210  -0.042   0.802   0.014   0.185
B L  4GU0E                 20    0.159  29.040  -0.389   0.879   0.010   0.111
E A  4GU0E                 21    0.332  36.630  -0.747   0.802   0.014   0.185
E T  4GU0E                 22    0.505  70.043  -0.174   0.802   0.014   0.185
E K  4GU0E                 23    0.555 114.122   0.072   0.717   0.014   0.269
E A  4GU0E                 24    0.458  50.416  -1.506   0.600   0.003   0.397
E A  4GU0E                 25    0.548  60.368  -1.449   0.406   0.004   0.590
E R  4GU0E                 26    0.848 194.146   0.373   0.003   0.003   0.994

Explain the output.  Go back.
```

---
